# Attenuated RORC Expression in the Presence of EMT Progression in Somatotroph Adenomas following Treatment with Somatostatin Analogs Is Associated with Poor Clinical Recovery

**DOI:** 10.1371/journal.pone.0066927

**Published:** 2013-06-25

**Authors:** Tove Lekva, Jens Petter Berg, Ansgar Heck, Stine Lyngvi Fougner, Ole Kristoffer Olstad, Geir Ringstad, Jens Bollerslev, Thor Ueland

**Affiliations:** 1 Section of Specialized Endocrinology, Department of Endocrinology, Oslo University Hospital, Oslo, Norway; 2 Research Institute for Internal Medicine, Oslo University Hospital, Oslo, Norway; 3 Faculty of Medicine, University of Oslo, Oslo, Norway; 4 Institute of Clinical Medicine, Oslo University Hospital, Oslo, Norway; 5 Department of Medical Biochemistry, Oslo University Hospital, Oslo, Norway; 6 Department of Endocrinology, St. Olavs Hospital, Trondheim University Hospital, Trondheim, Norway; 7 Department of Radiology and Nuclear Medicine, Oslo University Hospital, Oslo, Norway; University of Colorado, United States of America

## Abstract

Somatostatin analogs (SA) have been established as the first line medical treatment for acromegaly, but following long-term treatment, SA normalizes GH and IGF-I levels in only 40–60% of patients. The epithelial marker E-cadherin plays a crucial role in the epithelial mesenchymal transition (EMT) and is associated with a poor response to SA treatment. We hypothesized that the characterization of transcripts regulated by SA in somatotroph adenomas with high and low E-cadherin expression may identify signaling pathways and mediators that can explain the poor response to SA treatment. We performed a microarray analysis of sixteen adenomas with different levels of E-cadherin and SA treatment to identify regulated transcripts. Candidate transcripts were further explored *in vivo* in sixty-five adenomas, and interactions between SA treatment and EMT progression on mRNA expression profiles and associations with clinical recovery were assessed. Finally, the effects of SA treatment on adenoma cells *in vitro* from acromegalic patients were determined. Microarray analysis of selected adenomas with differential E-cadherin expression, as a marker of EMT progression, identified 172 genes that displayed differential expression that was dependent on SA treatment. The validation of selected candidates in the entire cohort identified 9 transcripts that showed an interaction between E-cadherin expression and SA treatment. Further analysis of the impact of these genes suggests that attenuated RORC expression in somatotroph adenomas is associated with increased tumor size and a blunted clinical response. Our study indicates that attenuated RORC may be involved in the poor clinical response to SA treatment in patients with acromegaly.

## Introduction

Somatostatin analogs (SA) have been established as the first line medical treatment for acromegaly and should result in the suppression of elevated blood GH and IGF-I levels and/or a significant tumor size reduction [1]. The response to SA depends on the presence and/or ratio of somatostatin receptor subtypes (SSTR_1–5_) on tumor cells; SSTR2, especially, has been positively correlated to the GH-lowering effect of hitherto clinically available analogs [2]–[5]. However, following long-term treatment, SA normalizes GH and IGF-I levels in only 40–60% of patients [6].

Previous studies in somatotroph adenomas suggest that attenuated E-cadherin expression, which is lost in the epithelial mesenchymal transition (EMT), is associated with reduced responsiveness to SA treatment as well as increased tumor size and invasiveness [7], [8]. E-cadherin has also been positively correlated with the SSTR2 protein receptor subtype [7]. Using microarray analysis, we have recently demonstrated that a large number of RNA transcripts are associated with E-cadherin expression in somatotroph adenomas and thus may be implicated in EMT progression in these tumors [8]. Further investigation of transcripts at different stages of EMT and the response to somatostatin analogs may identify signaling pathways and mediators that can explain the poor response to SA treatment. In the present study, the modulators of the poor response to SA treatment were explored through a microarray analysis of adenomas with different expression levels of E-cadherin, as a marker of EMT progression, to identify transcripts that were differentially expressed after SA treatment of tumors and that were associated with E-cadherin mRNA expression. The clinical importance of these transcripts was then investigated by correlating mRNA expression levels with clinical indices of disease activity and treatment response.

## Materials and Methods

### Patients and Samples

**Figure 1:**
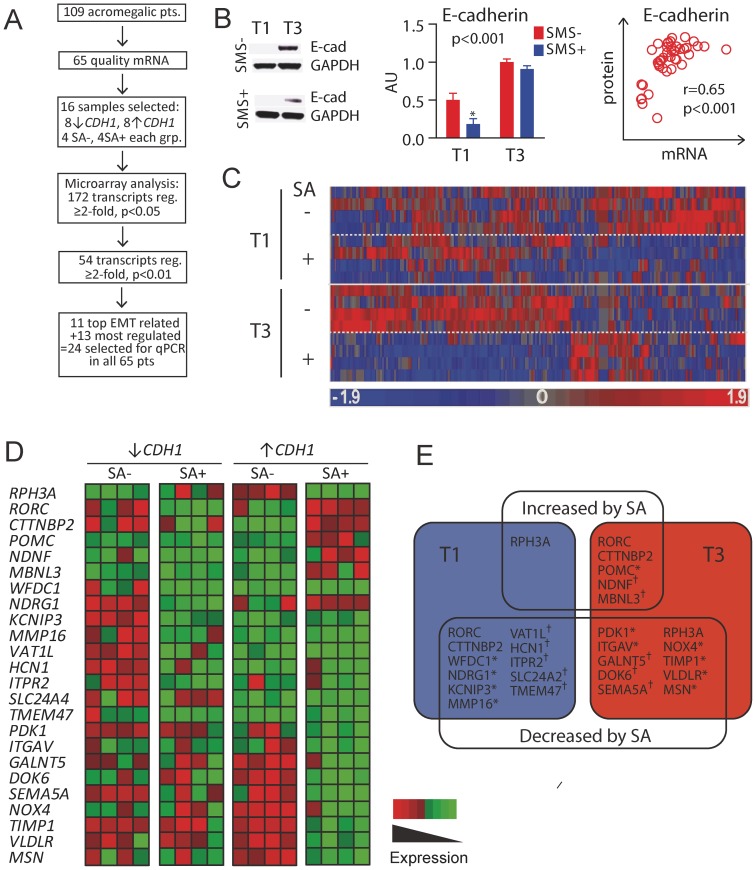
Study designs. (A) Overall study design. (B) Western blot of E-cadherin and GAPDH. Data for the E-cadherin protein ratio presented in the adenomas from E-cadherin gene expression tertile 1 and tertile 3. Correlation plot between E-cadherin protein and mRNA levels. (C) Gene expression profiles of differentially expressed genes in 16 adenomas selected from low and high tertiles of E-cadherin expression; 4 adenomas in each tertile were pretreated with SA. Gene expression levels for each adenoma are presented in horizontal rows with colors indicating upregulated (red) or downregulated (blue) genes. A total of 172 genes were differentially expressed in response to SA; 57 were downregulated and 1 was upregulated in tertile 1, while 93 were downregulated and 22 were upregulated in tertile 3 (mean fold change ≥2.0 and p<0.05). Each column represents a single gene. (D) A more detailed gene expression profile is presented for the 24 selected transcripts. The color intensity represents the degree of regulation; green indicates downregulation, while and red indicates upregulation. (E) Regulated SA transcripts presented. Transcripts were selected based on their known association with EMT or E-cadherin (*) or were chosen because of their strong regulation by the different levels of E-cadherin expression and by SA (†) (mean fold change ≥2.0 and p<0.01).

One hundred nine patients with active acromegaly, based on clinical evaluation and biochemical workup [7], [9], [10], who all underwent transsphenoidal pituitary surgery in the period from 1996 to 2011, were consecutively enrolled in the present study. Of these, sixty-five patients were included based on the availability of an adequate RNA specimen (Figure 1A) from the tumor as assessed with an Agilent 2100 Bioanalyzer (Agilent Technologies, Santa Clara, CA), of whom thirty-eight were untreated and twenty-seven were preoperatively treated with SA for 6 months (median). Table 1 provides an overview of the study population. The study was approved by the Regional Committee for Medical and Research Ethics, South-East, Norway, and was conducted according to the Declaration of Helsinki II. Written informed consent was obtained from all patients.

**Table 1:** Demographics of the study population.

Groups	Total	Direct surgery	Preoperative SA
	(n = 65)	(n = 38)	(n = 27)
Age (yr)	47 (41, 57)	49 (42, 56)	47 (37, 60)
Women/men (n)	28/37	16/22	12/15
Tumor size (cm^3^)	1.21 (0.54, 3.12)	0.68 (0.46, 2.08)†	2.19 (0.81, 5.40)
*Biochemistry*			
Serum GH (mU/L)	32 (20, 70)	28 (15, 46)†	53 (24, 108)
Serum IGF-I (nmol/L)	106 (83, 131)	96 (83, 125)	109 (90, 139)
*SMS response*			
GH reduction (%) n = 59#	88 (69, 92)	84.2 (62, 90)	89 (69, 95)
IGF-I reduction (%) n = 27*			54 (12, 62)
GH reduction (%) n = 27*			73 (41, 95)
Tumor size reduction (%) n = 26*			26 (5, 43)

Unless stated, data are given as median (25^th^, 75^th^ percentile).

† p<0.05 vs. Preoperative SA.

# During acute octreotide test in patients not preoperatively treated with a somatostatin analog;

* after median 6 months (range 2–32 months) preoperative SA treatment.

### Biochemical Measurements

Blood samples were drawn after an overnight fast, and the serum was isolated. Serum IGF-Ι levels were measured with a RIA (Nichols Institute, Nijmegen, The Netherlands) or Immulite 2000 (Siemens, Munich, Germany), and the mean daytime (3–5 times) GH (detection limit 0.3 mU/l) was measured by AutoDelfia (Wallac Oy, Turku, Finland) and, after 2005, by Immulite 2000 (Siemens) calibrated to the WHO standard IS 98/574. When the methods were changed, a cross-calibration was performed. An acute somatostatin test was performed in 59 patients prior to any treatment; the test measured the serum GH concentration before and 2–4 hours after the test dose [7]. The relative reduction was calculated by comparing the mean GH values before the injection with the mean GH levels measured 2–4 hours after the test dose [2].

### Estimation of Tumor Size by MRI

The formula of width×height×length×0.5 was used by a neuroradiologist to estimate the size of each tumor. For the patients treated primarily with SA, MRI scans were available for twenty-six patients before and after SA treatment.

### Microarray Analysis

The methods used for the analysis of E-cadherin mRNA levels and the subsequent microarray analysis in selected adenomas have been previously reported [8]. Briefly, based on the E-cadherin mRNA expression determined by quantitative real-time RT-PCR (RT-qPCR in all 65 adenomas), mRNA from 16 patients, 8 with the lowest E-cadherin expression (from tertile 1), of which 4 were pretreated with SA, and 8 with the highest E-cadherin expression (from tertile 3), of which 4 pretreated with SA, was chosen for microarray analysis (Figure 1A). As reported [8], 100 ng of total RNA was processed with a GeneChip HT One-Cycle cDNA Synthesis Kit and a GeneChip HT IVT Labeling Kit, following the manufacturer’s protocol for whole genome gene expression analysis (Affymetrix, Santa Clara, CA, USA). Labeled and fragmented single stranded cDNAs were hybridized to the GeneChip Human Gene 1.0 ST Arrays (28869 transcripts) (Affymetrix). The arrays were washed and stained using a FS-450 fluidics station (Affymetrix). Signal intensities were detected with a Hewlett Packard Gene Array Scanner 3000 7G (Hewlett Packard, Palo Alto, CA, USA). The scanned images were processed using the Affymetrix GeneChip Command Console (AGCC). The CEL files were imported into the Partek Genomics Suite software (Partek, Inc. MO, USA). Robust microarray analysis (RMA) was applied for normalization. The cluster analysis was generated in the Partek Genomics Suite. All raw microarray data have been deposited in the Gene Expression Omnibus (GEO) database under the accession number GSE46311.

### Protein Extraction and Western Blot Analysis

The adenoma tissue was frozen at −70°C shortly after pituitary surgery. The tissue was homogenized in TRIzol reagent (Invitrogen Corp., Carlsbad, CA), and the protein was extracted following the manufacturer’s instructions. The proteins were precipitated and washed; the protein concentration was then measured, and the Western blot performed as previously described [7] with 15 µg of total protein applied per lane. The blots were probed with a mouse monoclonal anti-E-cadherin antibody (1∶1000, ab1416; Abcam PLC, Cambridge, UK), mouse monoclonal antiglyceraldehyde-3-phosphate dehydrogenase (GAPDH) antibody (1∶10,000, G8795; Sigma-Aldrich Corp., St. Louis, MO) and secondary antibody antimouse IgG (1∶10 000, Jackson ImmunoResearch Laboratories Europe, Suffolk, UK), as previously described. The Multi Gauge software (Fuji film Corp., Tokyo, Japan) was used for the data analysis of the protein levels. The band signal for each antibody was adjusted for background. The E-cadherin to GAPDH ratio for each adenoma was calculated (E-cadherin ratio) and used as a measure of the E-cadherin protein level.

### RNA Isolation and RT-qPCR

The extraction of total RNA was performed using Trizol (Invitrogen, Carlsbad). The RNA was purified using a QIAGEN RNeasy micro kit (Qiagen, Valencia, CA). The integrity was assessed using an Agilent 2100 Bioanalyzer (Agilent Technologies, Santa Clara, CA ), and the concentrations were determined by OD readings on a Nanodrop ND-1000 Spectrophotometer (Nanodrop Technologies,Wilmington, DE). Reverse transcription was performed using a High Capacity cDNA Archive Kit (Applied Biosystems, Foster City, CA). The mRNA quantification was performed using the standard curve method of the ABI Prism 7500 (Applied Biosystems). For the RT-qPCR, sequence specific exon-exon spanning oligonucleotide primers were designed using the Primer Express software version 2.0 (Applied Biosystems), (Table S1). The transcript expression levels were normalized to the GAPDH mRNA levels in human pituitary samples and expressed as relative mRNA levels (data were log transformed to normal distribution in human pituitary adenomas).

### Octreotide Treatment of Adenoma Cells from Acromegalic Patients *in vitro*

Adenoma tissue from five patients was used for the primary culture to examine the *in vitro* effects of octreotide treatment, as previously reported [7]. Approximately 1×10^5^ cells/well were cultured with and without 10^−8^ M octreotide for 6 and 24 hours, in quadruplicate. Two of the adenomas had an E-cadherin mRNA expression level 300 times lower than the other three. The expression levels of RORC was normalized to the β-actin mRNA levels and expressed as relative mRNA levels. All the adenomas had been preoperatively treated with SA except for one adenoma.

### Statistics

Differences in the patient demographics were analyzed with the Mann-Whitney U-test. The transcripts analyzed in all patients by RT-qPCR were not normally distributed and were log transformed prior to the regression analysis. The interaction between E-cadherin and SA treatment was analyzed by univariate regression with the transcripts as the dependent variable and the E-cadherin level, treatment with SA and the interaction term between E-cadherin and SA treatment (log E-cadherin*SA treatment) as covariates by direct entry. Stepwise linear regression was performed to identify transcripts that were predictors of tumor size and the response to the acute SA test as well as tumor size- and IGF-1-reduction following SA treatment. A two-sided p value <0.05 was considered significant, except when analyzing the effect of the interaction term, when p<0.1 was considered significant. Differentially expressed genes between groups were identified using one-way ANOVA of the microarray data. An unpaired Student`s sample t-test (two-tailed) was used to evaluate the differences between untreated and octreotide-treated adenoma cells in the *in vitro* studies.

## Results

The clinical characteristics of the study population are given in Table 1. Patients receiving preoperative SA were characterized by higher serum GH levels and larger tumor sizes. No other differences between the two groups were detected.

### Microarray

To screen for differentially expressed transcripts in relation to the E-cadherin expression levels and SA treatment, total RNA from sixteen of the pituitary GH adenomas, as described above, with the highest and lowest E-cadherin expression (i.e., 8 of each, of which 4 were pretreated with SA, figure 1A) levels as determined by RT-PCR, were subjected to whole-genome gene expression profiling as reported previously [8]. The mRNA levels were associated with previously published E-cadherin protein levels [7] from 45 adenomas (r = 0.65, p<0.001) (Figure 1B), presented by western blot in two of the adenomas, and the E-cadherin protein ratio from the adenomas in tertile 1 and 3 are included in the graph (Figure 1B). Our primary goal was to identify potentially interesting transcripts that could be validated in the total patient population (n = 65) (Figure 1A) and that were correlated to disease activity and indices of clinical recovery following SA treatment. First, we compared transcripts that were differentially expressed in adenomas that were or were not pretreated with SA and identified 172 mRNA transcripts regulated by SA in the high and low E-cadherin tertiles (regulated ≥2.0-fold and p<0.05 by SA in tertile 1 and/or tertile 3, Figure 1C). Second, we were primarily interested in the transcripts that were differentially expressed after SA treatment in relation to E-cadherin expression. Transcripts regulated in the same direction in the high and low E-cadherin tertiles were therefore excluded. When considering adenomas with low E-cadherin expression (i.e., tertile 1), 57 genes showed decreased expression and 1 gene showed increased expression in adenomas from patients pre-treated with SA. In adenomas with high E-cadherin expression (i.e., tertile 3), 93 genes showed decreased expression and 22 genes showed increased expression in the SA- treated adenomas compared with the untreated adenomas. To narrow the list for further analyses, we selected transcripts that were regulated with a p-value <0.01, which reduced the total number of transcripts from 172 to 54. Of these 54 regulated transcripts, 11 had been described in the literature as being associated with the EMT process or E-cadherin expression [11]–[23], and the remaining 13 were chosen because of their strong regulation by the different levels of E-cadherin induced by SA (Figure 1D–E).

### RT-qPCR

**Figure 2:**
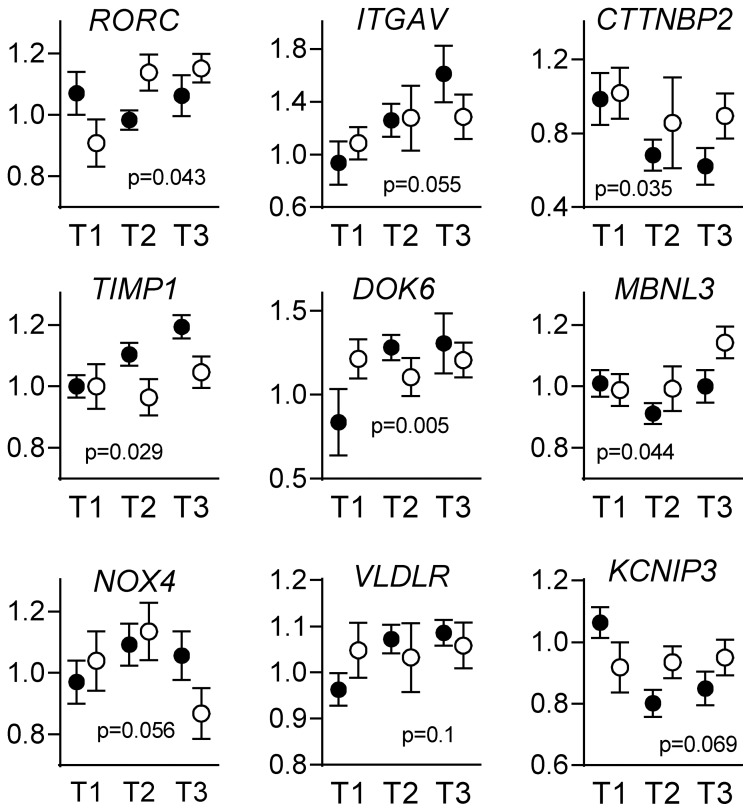
Quantitative real time RT-PCR of selected gene transcripts in somatotroph adenomas, which supported an interaction between E-cadherin expression levels and SA treatment. The transcripts of (A) RORC (B) ITGAV (C) CTTNBP2 (D) TIMP1 (E) DOK6 (F) MBNL3 (G) NOX4 (H) VLDLR and (I) KCNIP3 in 65 growth hormone-producing adenomas were selected based on the interaction between E-cadherin and SA (p<0.1, interaction tested with continuous E-cadherin, but presented as tertiles), quantified and distributed according to tertiles of E-cadherin mRNA expression (normalized to GAPDH mRNA levels and expressed as relative mRNA levels). The results are presented as the mean ± SEM and presented relative to tertile 1. P-values represent the interaction between E-cadherin and SA in the univariate analysis.

To verify the interaction between tumor E-cadherin expression and SA treatment, the expression of the selected 24 transcripts were quantified by RT-qPCR in the initial 65 GH-producing adenomas. The interaction between SA treatment and E-cadherin was assessed by univariate regression, as described in the methods section. As presented in Figure 2, which shows the individual genes according to E-cadherin tertiles and treatment with SA, the interaction term was significant (p<0.1) for 9 transcripts: RAR-related orphan receptor C (*RORC*) (p = 0.043), integrin alpha V (*ITGAV*) (p = 0.055), cortactin binding protein 2 (*CTTNBP2*) (p = 0.035), TIMP metallopeptidase inhibitor 1 (*TIMP1*) (p = 0.029), docking protein 6 (*DOK6*) (p = 0.005), muscleblind-like splicing regulator 3 (*MBNL3*) (p = 0.044), NADPH oxidase 4 (*NOX4*) (p = 0.056), very low density lipoprotein receptor (*VLDLR*) (p = 0.100) and Kv channel interacting protein 3, which is also known as calsenilin (*KCNIP3*) (p = 0.069). The other transcripts, which were not associated with a significant interaction between E-cadherin and SA, are presented in Figure S1. Note that the results of an interaction analysis could confirm an interaction between the E-cadherin and SA treatment, but it is not a validation of the array per se. The expression levels of transcripts quantified by the microarray and qPCR in the 16 adenomas were highly correlated (median r = 0.83), and the low number of validated transcripts is not a technical issue. However, 3 transcripts correlated poorly, possibly due to the presence of different isoforms or low expression (i.e., qPCR CTs >30 cycles).

### Associations between Selected Transcripts and Clinical Variables

**Figure 3:**
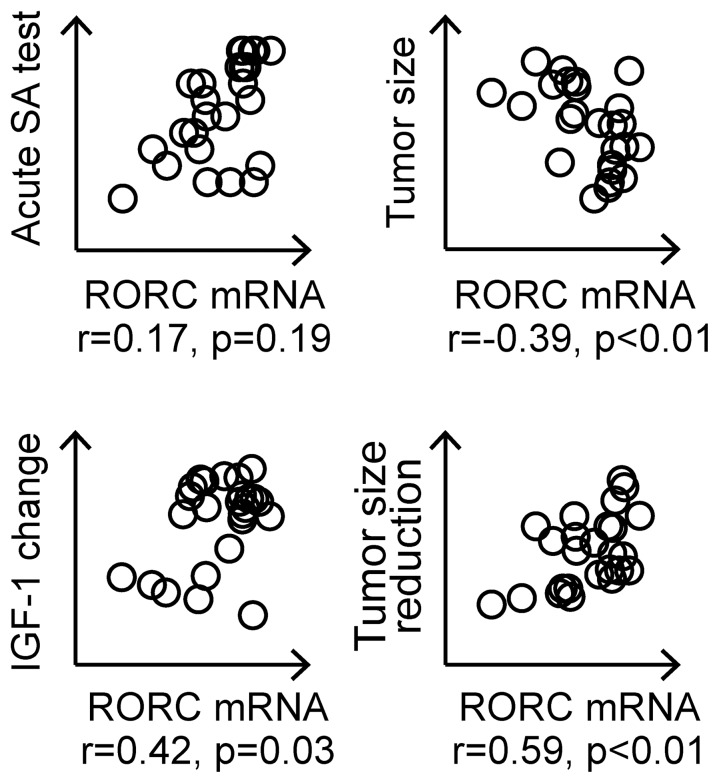
Matrix scatter plots between *RORC* and the response to the acute SA test, tumor size, tumor size reduction and IGF-I reduction.

The main goal of treating acromegaly is to normalize GH and IGF-I levels and to reduce or control tumor volume. When correlating the transcripts that were verified in the total patient population with important clinical parameters, including tumor size, acute somatostatin test, reduction in IGF-I levels (%) and tumor size reduction (%), several significant correlations were found (Table 2). In addition to the transcripts chosen, we also included *SSTR2* mRNA levels as an independent variable because of this gene’s well-known association with response to treatment [2]–[5]. We first investigated associations between the mRNA expression levels of the selected transcripts and the response to the acute SA test (n = 59) and tumor size (n = 64). Some of the patients had SA treatment before tumor tissue was collected, and SA treatment was therefore included in the regression analysis by direct entry with the other variables included in the stepwise linear regression. Univariate analysis identified a significant correlation between *SSTR2* and the response to the acute SA test, while SA treatment, *RORC* and *DOK6* mRNA levels were correlated with tumor size. Multiple stepwise linear regression identified *SSTR2*, *TIMP1* and *KCNIP3* as predictors of the response to the acute SA test and SA treatment and *RORC* as a predictor of tumor size (Table 2). We subsequently investigated the associations between percentage tumor size and IGF1-I reduction and mRNA expression of the selected transcripts. Twenty-seven patients received SA treatment prior to surgery. In these patients, the percentage tumor size and IGF-I reduction were measured as previously reported [8]. Univariate analysis indicated that the elevated mRNA levels of both *SSTR2* and *RORC* were associated with a larger decrease in IGF-1 levels and tumor reduction. These results were largely confirmed in multiple analyses where stepwise linear regression identified *RORC* and *KCNIP3* as significant predictors of percentage tumor size reduction. For reduction in circulating IGF-I, *SSTR2*, *KCNIP3* and *RORC* were identified as significant predictors (Table 2). The association between the RORC mRNA levels and clinical variables are presented in Figure 3.

**Table 2:** Associations between selected transcipts and clinical variables.

	Acute somatostatin test		Tumor size		% reduction IGF-1		% reduction tumor size	
	Univariate^a^	Multivariate^b^	Univariate^a^	Multivariate^b^	Univariate^a^	Multivariate^b^	Univariate^a^	Multivariate^b^
SA treatment	0.22 (0.10)	1.01 (0.68), t = 1.49,p = 0.14	0.30 (0.02)	0.72 (0.25),t = 2.83, p = <0.01	NA	NA	NA	NA
SSTR2	0.37(<0.01)	1.35 (0.35), t = 3.86,p<0.01	−0.21 (0.10)		0.52 (<0.01)	10.90 (4.42), t = 2.46, p = 0.02	0.42 (0.03)	
TIMP1	−0.23 (0.08)	−1.08 (0.42), t = −2.61, p = 0.01	−0.07 (0.58)		−0.25 (0.21)		−0.31 (0.13)	
KCNIP3	−0.15 (0.26)	−0.52 (0.26), t = −2.00, p = 0.05	−0.02 (0.85)		−0.27 (0.17)	−9.62 (3.48), t = −2.76, p = 0.01	−0.12 (0.56)	−10.74 (4.26), t = −2.52, p = 0.02
RORC	0.17 (0.19)		−0.39 (<0.01)	−0.38 (0.10), t = −3.94, p = <0.01	0.42 (0.03)	7.50 (3.54), t = 2.11, p = 0.05	0.59 (<0.01)	14.62 (3.84), t = 3.81, p = <0.01
ITGAV	−0.08 (0.55)		−0.18 (0.15)		−0.02 (0.91)		−0.05 (0.80)	
CTTNBP2	0.02 (0.91)		−0.08 (0.51)		0.14 (0.48)		0.06 (0.76)	
DOK6	−0.03 (0.80)		−0.28 (0.03)	−0.13 (0.07), t = −1.74, p = 0.09	0.05 (0.82)		−0.09 (0.64)	
MBNL3	0.24 (0.07)		−0.09 (0.49)		0.36 (0.06)		0.35 (0.08)	
NOX4	−0.14 (0.28)		−0.20 (0.11)	−0.13 (0.09), t = −1.53, p = 0.131	−0.17 (0.40)		−0.35 (0.09)	−4.88 (3.12), t = −1.56, p = 0.13
VLDLR	−0.14 (0.29)		0.06 (0.66)		−0.02 (0.93)		−0.30 (0.14)	
R Square		0.31		0.35		0.51		0.52

a Univariate, Pearson correlation: r (p = ). **^b^**Multivariate, Stepwise linear regression: B (SE), t = , p = . NA, not applicable.

### Association between E-cadherin Expression and Response to Octreotide in Tumors *in vitro*

**Figure 4:**
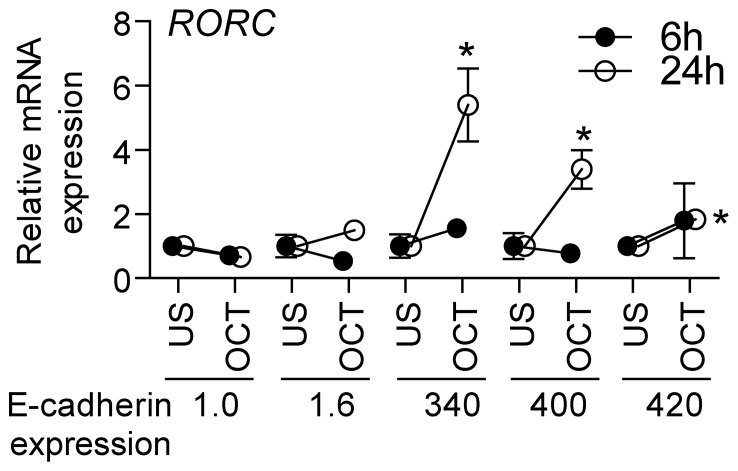
Octreotide treatment of human pituitary GH tumors from acromegalic patients. Gene expression of (A) RORC following 6 and 24 h of treatment with octreotide (10^−8^ M) in quadruplicate, normalized to β-actin and expressed as relative mRNA levels. There were two of the adenomas that had an E-cadherin mRNA expression level 300 times lower than the other three, in the adenoma tissue before any culturing. The results are from five independent experiments and presented as the mean ± SEM, * *p*<0.05 indicating difference between untreated control and octreotide-treated cells normalized to untreated control at each time point.

We next analyzed RORC mRNA expression in freshly prepared adenoma tissues that had been cultured *in vitro* and treated with octreotide. The adenomas were divided into two groups according to their E-cadherin mRNA expression levels from the adenoma tissue. We found RORC to be upregulated by octreotide treatment for 24 h in the adenomas with high E-cadherin expression, while no change or a minor decrease was observed in adenomas with low E-cadherin expression (Figure 4).

## Discussion

In the present study, we investigated interactions between SA treatment and EMT progression on mRNA expression profiles in somatotroph adenomas and associations with clinical recovery.

Our main and novel findings were as follows: *i)* Microarray analysis of selected adenomas with different E-cadherin expression levels, as a marker of EMT progression, identified 172 genes that displayed differential expression dependent on SA treatment. *ii)* Validation of selected candidates in the entire cohort identified 9 transcripts that showed an interaction between E-cadherin expression and SA treatment response. *iii)* Further analysis of the impact of these genes suggests that attenuated RORC expression in somatotroph adenomas is associated with an increased tumor size and a blunted clinical response following SA treatment.

We have previously shown that increased E-cadherin mRNA levels in pituitary tumor tissue is associated with an enhanced response to SA treatment [8], supporting an interaction between SA response and EMT progression. In this study, we further explored this interaction in somatotroph adenomas and identified a large number of transcripts that displayed a differential mRNA expression depending on treatment with SA and E-cadherin mRNA levels, and 9 of 24 transcripts were validated (see discussion on validation below). The EMT-related genes that supported an interaction between E-cadherin and SA treatment were the transcripts involved in TGFβ cell migration and metastasis: *ITGAV* and *NOX4* [21], [24], which are a cell surface receptor [21] and a redox mediated signaling transcript, respectively [25]. Furthermore, *VLDLR* has been shown to be involved in fibrin-induced leukocyte transmigration, while *KCNIP3* may regulate inflammation through mechanisms involving an interaction with VE-cadherin and N-cadherin processing [17], [23]. *TIMP1*, a regulator of extracellular matrix degradation with proliferatory and antiapoptotic functions, has been shown to induce the expression of several EMT transcription factors [18]. The nuclear receptor *RORC* is involved in the regulation of circadian behaviors and clock gene expression [26], and it has also been implicated in lymphoma formation [27]. *CTTNBP2* may indirectly regulate cellular invasiveness [28]. The last two transcripts that supported an interaction with E-cadherin and SA treatment were *DOK6* and *MBNL3*, which are involved in promoting neurite outgrowth and alternative splicing [29], [30], respectively. SA is known to inhibit intracellular Ca^2+^ via the SSTR2 receptor and thereby reduced the release of GH [31]. The SSTR2 content in the tumor is a well-known contributor to the response to treatment [2], [32], and we confirmed that increased *SSTR2* mRNA expression is associated with a good response to the acute SA test. The multiple analyses identified several covariates that were not associated with the dependent variable in the univariate analysis (e.g., *TIMP1* and *KCNIP3* for response to the acute SA test; *KCNIP3* for the % reduction in IGF-1 and tumor reduction), and the impact of mRNA expression on these responses should be interpreted with caution. However, it is tempting to speculate that the indirect regulation of KCNIP3 transcript levels by SA could contribute to the short- and long-term clinical responses through EMT-related mechanisms, such as N-cadherin processing and calcium signaling [17], [33]. Although further studies of these transcripts may reveal important information about the pathology of SA treatment and EMT progression in somatotroph adenomas, only *RORC* was associated with tumor size prior to SA treatment, as well as tumor reduction and IGF-I reduction following SA treatment, both in the univariate and multiple analyses.

The *RORC* gene consists of two isoforms, γ1 and γ2 (γt); *RORγ1*, the isoform regulated in the microarray analysis of the somatotroph adenomas, is expressed in many tissues [27]. RORC exhibits an oscillatory expression pattern in different tissues, and mice deficient in the expression of RORC exhibit a high incidence of thymic lymphomas that metastasize frequently to the liver and spleen [27]. The loss of *ROR γ1* is shown to affect the peak expression of several clock genes [26], some of which are important for tumor progression [34], [35]. Our *in vivo* findings in somatotroph adenomas demonstrating a correlation between low *RORC* mRNA expression and increased tumor size support the biological significance of attenuated *RORC* expression in somatotroph adenomas. Furthermore, this low RORC expression levels could be a particularly unfavorable effect of SA treatment in patients with attenuated E-cadherin expression. In contrast, increased *RORC* expression was associated with SA treatment in patients with high pituitary E-cadherin mRNA levels. Thus, attenuated *RORC* expression was correlated with a blunted tumor size- and IGF-1 reduction in patients treated with SA, indicating that these analogs may adversely affect clinical recovery in the presence of EMT progression (i.e., low E-cadherin) involving *RORC*-mediated mechanisms. These observations were supported *in vitro* in primary cultures treated with octreotide; tumors characterized by low E-cadherin expression displayed an attenuated effect on RORC expression, while cultures from tumors with high E-cadherin expression showed an increase in *RORC* mRNA levels.

Some limitations of the study should be mentioned. Of the 24 transcripts selected for further verification in the whole cohort, only 9 confirmed an interaction between SA treatment and E-cadherin. In contrast, our previous study [8] investigating transcripts associated with E-cadherin expression, which used a similar approach, confirmed nearly all candidates in the whole cohort by qPCR. As discussed, this discrepancy was not a technical issue but could instead be due partly to the more straightforward approach in the previous study (i.e., 2×2 *vs.* 4×4) and the number of observations in each group (n = 8 *vs.* n = 4), making confirmation more likely. Furthermore, the inability to confirm the findings for more transcripts could also reflect the heterogeneous nature of pituitary tumors and response to treatment. Additionally, there are some inherent biases in our study design that should be mentioned. Our findings may partly be influenced by different tumor phenotypes because a massively invasive adenoma is more likely to receive medical treatment before operation than a small, resectable adenoma. However, the numbers of SA-treated adenomas in the different tertiles of E-cadherin expression were similar. It could also be argued that an *a priori* separation of low *vs.* high E-cadherin will identify surrogate markers. Thus, future functional studies are needed to evaluate and confirm the importance of the candidate transcripts and mechanisms involved. Most of the adenomas cultured *in vitro* had been treated with SA before surgery, and we cannot exclude that this treatment may have interfered with the *in vitro* experiments. Finally, although we were able to detect RORC protein by western blot in some adenomas with very high total protein levels, the amount of protein was in general too low to reliably detect RORC protein expression in the *in vivo* adenoma as well as in the primary cultures *in vitro* (data not shown).

Pituitary somatotroph adenomas are heterogeneous and have been classified into multiple subtypes. It has been argued that the loss of E-cadherin, while associated with the loss of epithelial differentiation, is not always indicative of EMT, as not all adenohypophysial cells express E-cadherin at the same level. However, it has been suggested recently that, within the EMT, the epithelial and mesenchymal cells can be regarded as the two extremes, indicating that intermediate phenotypes may correspond to partial EMTs [36]. Furthermore, the loss of adherens junctions and subsequent E-cadherin repression is a hallmark of EMT, and investigating associations between E-cadherin mRNA levels and candidates identified by microarray as continuous variables in the entire cohort in relation to clinical variables may therefore take into account the impact of partial EMT on these outcomes, which we believe is a strength of our study.

This study identified several transcripts in somatotroph adenomas that were differentially expressed after SA treatment depending on E-cadherin mRNA levels. In particular, adenomas with low E-cadherin expression was associated with decreased RORC expression following SA treatment and a blunted clinical response as demonstrated by attenuated IGF-1 and tumor size reduction. Further studies are needed to elucidate the interaction between EMT progression and RORC expression as well as the mechanism by which SA treatment may adversely affect this interaction and lead to poor clinical responses in patients with acromegaly.

## Supporting Information

Figure S1 **Quantitative real time RT-PCR of selected gene transcripts in somatotrophinomas with different E-cadherin expression levels and SA pretreatment, with no significant interaction between E-cadherin and SA.** The transcripts of (A) WFDC1 (B) NDRG1 (C) MMP16 (D) MSN (E) PDK1 (F) POMC (G) RPH3A (H) SLC24A2 (I) HCN1 (J) VAT1L (K) ITPR2 (L) SEMA5A (M) NDNF (N) GALNT5 and (O) TMEM47 in 65 growth hormone producing adenomas quantitated and distributed according to tertiles of E-cadherin mRNA expression (normalized to GAPDH mRNA levels and expressed as relative mRNA levels) and SA treatment. Results are presented as mean ± SEM and presented relative to tertile 1.
(TIF)

Table S1 **Primer sequences used in PCR reactions.**
(DOCX)

## References

[1] Melmed S, Colao A, Barkan A, Molitch M, Grossman AB, et al (2009) Guidelines for acromegaly management: an update. J Clin Endocrinol Metab 94: 1509–1517. 19208732 10.1210/jc.2008-2421

[2] Fougner SL, Borota OC, Berg JP, Hald JK, Ramm-Pettersen J, et al (2008) The clinical response to somatostatin analogues in acromegaly correlates to the somatostatin receptor subtype 2a protein expression of the adenoma. Clin Endocrinol (Oxf) 68: 458–465. 17941904 10.1111/j.1365-2265.2007.03065.x

[3] Hofland LJ, van der HJ, van Koetsveld PM, de Herder WW, Waaijers M, et al (2004) The novel somatostatin analog SOM230 is a potent inhibitor of hormone release by growth hormone- and prolactin-secreting pituitary adenomas in vitro. J Clin Endocrinol Metab 89: 1577–1585. 15070915 10.1210/jc.2003-031344

[4] Saveanu A, Gunz G, Dufour H, Caron P, Fina F, et al (2001) Bim-23244, a somatostatin receptor subtype 2- and 5-selective analog with enhanced efficacy in suppressing growth hormone (GH) from octreotide-resistant human GH-secreting adenomas. J Clin Endocrinol Metab 86: 140–145. 11231991 10.1210/jcem.86.1.7099

[5] Taboada GF, Luque RM, Neto LV, Machado EO, Sbaffi BC, et al (2008) Quantitative analysis of somatostatin receptor subtypes (1–5) gene expression levels in somatotropinomas and correlation to in vivo hormonal and tumor volume responses to treatment with octreotide LAR. Eur J Endocrinol 158: 295–303. 18299461 10.1530/EJE-07-0562

[6] Sherlock M, Woods C, Sheppard MC (2011) Medical therapy in acromegaly. Nat Rev Endocrinol 7: 291–300. 21448141 10.1038/nrendo.2011.42

[7] Fougner SL, Lekva T, Borota OC, Hald JK, Bollerslev J, et al (2010) The expression of E-cadherin in somatotroph pituitary adenomas is related to tumor size, invasiveness, and somatostatin analog response. J Clin Endocrinol Metab 95: 2334–2342. 20335450 10.1210/jc.2009-2197

[8] Lekva T, Berg JP, Fougner SL, Olstad OK, Ueland T, et al (2012) Gene Expression Profiling Identifies ESRP1 as a Potential Regulator of Epithelial Mesenchymal Transition in Somatotroph Adenomas from a Large Cohort of Patients with Acromegaly. J Clin Endocrinol Metab 97: E1506–E1514. 22585092 10.1210/jc.2012-1760

[9] Fougner SL, Casar-Borota O, Heck A, Berg JP, Bollerslev J (2012) Adenoma granulation pattern correlates with clinical variables and effect of somatostatin analogue treatment in a large series of patients with acromegaly. Clin Endocrinol (Oxf) 76: 96–102. 21722151 10.1111/j.1365-2265.2011.04163.x

[10] Heck A, Ringstad G, Fougner SL, Casar-Borota O, Nome T, et al (2012) Intensity of pituitary adenoma on T2-weighted magnetic resonance imaging predicts the response to octreotide treatment in newly diagnosed acromegaly. Clin Endocrinol (Oxf) 77: 72–78. 22066905 10.1111/j.1365-2265.2011.04286.x

[11] Delassus GS, Cho H, Hoang S, Eliceiri GL (2010) Many new down- and up-regulatory signaling pathways, from known cancer progression suppressors to matrix metalloproteinases, differ widely in cells of various cancers. J Cell Physiol 224: 549–558. 20432456 10.1002/jcp.22157

[12] Feng Q, Di R, Tao F, Chang Z, Lu S, et al (2010) PDK1 regulates vascular remodeling and promotes epithelial-mesenchymal transition in cardiac development. Mol Cell Biol 30: 3711–3721. 20457809 10.1128/MCB.00420-10 PMC2897563

[13] Haynes J, Srivastava J, Madson N, Wittmann T, Barber DL (2011) Dynamic actin remodeling during epithelial-mesenchymal transition depends on increased moesin expression. Mol Biol Cell 22: 4750–4764. 22031288 10.1091/mbc.E11-02-0119 PMC3237619

[14] Hellman NE, Spector J, Robinson J, Zuo X, Saunier S, et al (2008) Matrix metalloproteinase 13 (MMP13) and tissue inhibitor of matrix metalloproteinase 1 (TIMP1), regulated by the MAPK pathway, are both necessary for Madin-Darby canine kidney tubulogenesis. J Biol Chem 283: 4272–4282. 18039671 10.1074/jbc.M708027200

[15] Himes AD, Fiddler RM, Raetzman LT (2011) N-cadherin loss in POMC-expressing cells leads to pituitary disorganization. Mol Endocrinol 25: 482–491. 21273444 10.1210/me.2010-0313 PMC3045739

[16] Iiizumi M, Liu W, Pai SK, Furuta E, Watabe K (2008) Drug development against metastasis-related genes and their pathways: a rationale for cancer therapy. Biochim Biophys Acta 1786: 87–104. 18692117 10.1016/j.bbcan.2008.07.002 PMC2645343

[17] Jang C, Choi JK, Na YJ, Jang B, Wasco W, et al (2011) Calsenilin regulates presenilin 1/gamma-secretase-mediated N-cadherin epsilon-cleavage and beta-catenin signaling. FASEB J 25: 4174–4183. 21852538 10.1096/fj.11-185926

[18] Jung YS, Liu XW, Chirco R, Warner RB, Fridman R, et al.. (2012) TIMP-1 Induces an EMT-Like Phenotypic Conversion in MDCK Cells Independent of Its MMP-Inhibitory Domain. PLoS One 7: e38773–. 10.1371/journal.pone.0038773 PMC3372473 22701711

[19] Kachhap SK, Faith D, Qian DZ, Shabbeer S, Galloway NL, et al.. (2007) The N-Myc down regulated Gene1 (NDRG1) Is a Rab4a effector involved in vesicular recycling of E-cadherin. PLoS One 2: e844–. 10.1371/journal.pone.0000844 PMC1952073 17786215

[20] McAlhany SJ, Ayala GE, Frolov A, Ressler SJ, Wheeler TM, et al (2004) Decreased stromal expression and increased epithelial expression of WFDC1/ps20 in prostate cancer is associated with reduced recurrence-free survival. Prostate 61: 182–191. 15305341 10.1002/pros.20085

[21] Sun H, Hu K, Wu M, Xiong J, Yuan L, et al (2011) Contact by melanoma cells causes malignant transformation of human epithelial-like stem cells via alpha V integrin activation of transforming growth factor beta1 signaling. Exp Biol Med (Maywood ) 236: 352–365. 21427239 10.1258/ebm.2010.010106

[22] Xiao L, Ge Y, Sun L, Xu X, Xie P, et al (2012) Cordycepin inhibits albumin-induced epithelial-mesenchymal transition of renal tubular epithelial cells by reducing reactive oxygen species production. Free Radic Res 46: 174–183. 22149621 10.3109/10715762.2011.647688

[23] Yakovlev S, Mikhailenko I, Cao C, Zhang L, Strickland DK, et al (2012) Identification of VLDLR as a novel endothelial cell receptor for fibrin that modulates fibrin-dependent transendothelial migration of leukocytes. Blood 119: 637–644. 22096238 10.1182/blood-2011-09-382580 PMC3257021

[24] Boudreau HE, Casterline BW, Rada B, Korzeniowska A, Leto TL (2012) Nox4 involvement in TGF-beta and SMAD3-driven induction of the epithelial-to-mesenchymal transition and migration of breast epithelial cells. Free Radic Biol Med 53: 1489–1499. 22728268 10.1016/j.freeradbiomed.2012.06.016 PMC3448829

[25] Ushio-Fukai M, Nakamura Y (2008) Reactive oxygen species and angiogenesis: NADPH oxidase as target for cancer therapy. Cancer Lett 266: 37–52. 18406051 10.1016/j.canlet.2008.02.044 PMC2673114

[26] Takeda Y, Jothi R, Birault V, Jetten AM (2012) RORgamma directly regulates the circadian expression of clock genes and downstream targets in vivo. Nucleic Acids Res 40: 8519–8535. 22753030 10.1093/nar/gks630 PMC3458568

[27] Jetten AM (2009) Retinoid-related orphan receptors (RORs): critical roles in development, immunity, circadian rhythm, and cellular metabolism. Nucl Recept Signal 7: e003–. 10.1621/nrs.07003 PMC2670432 19381306

[28] Chen YK, Hsueh YP (2012) Cortactin-binding protein 2 modulates the mobility of cortactin and regulates dendritic spine formation and maintenance. J Neurosci 32: 1043–1055. 22262902 10.1523/JNEUROSCI.4405-11.2012 PMC6621164

[29] Ho TH, Charlet B, Poulos MG, Singh G, Swanson MS, et al (2004) Muscleblind proteins regulate alternative splicing. EMBO J 23: 3103–3112. 15257297 10.1038/sj.emboj.7600300 PMC514918

[30] Li W, Shi L, You Y, Gong Y, Yin B, et al.. (2010) Downstream of tyrosine kinase/docking protein 6, as a novel substrate of tropomyosin-related kinase C receptor, is involved in neurotrophin 3-mediated neurite outgrowth in mouse cortex neurons. BMC Biol 8: 86–. 10.1186/1741-7007-8-86 PMC2901200 20565848

[31] Petrucci C, Cervia D, Buzzi M, Biondi C, Bagnoli P (2000) Somatostatin-induced control of cytosolic free calcium in pituitary tumour cells. Br J Pharmacol 129: 471–484. 10711345 10.1038/sj.bjp.0703075 PMC1571859

[32] Casarini AP, Jallad RS, Pinto EM, Soares IC, Nonogaki S, et al (2009) Acromegaly: correlation between expression of somatostatin receptor subtypes and response to octreotide-lar treatment. Pituitary 12: 297–303. 19330452 10.1007/s11102-009-0175-1

[33] Sours-Brothers S, Ma R, Koulen P (2009) Ca2+-sensitive transcriptional regulation: direct DNA interaction by DREAM. Front Biosci 14: 1851–1856. 10.2741/3346 19273168

[34] Grechez-Cassiau A, Rayet B, Guillaumond F, Teboul M, Delaunay F (2008) The circadian clock component BMAL1 is a critical regulator of p21WAF1/CIP1 expression and hepatocyte proliferation. J Biol Chem 283: 4535–4542. 18086663 10.1074/jbc.M705576200

[35] Takeda Y, Kang HS, Angers M, Jetten AM (2011) Retinoic acid-related orphan receptor gamma directly regulates neuronal PAS domain protein 2 transcription in vivo. Nucleic Acids Res 39: 4769–4782. 21317191 10.1093/nar/gkq1335 PMC3113563

[36] Jordan NV, Johnson GL, Abell AN (2011) Tracking the intermediate stages of epithelial-mesenchymal transition in epithelial stem cells and cancer. Cell Cycle 10: 2865–2873. 21862874 10.4161/cc.10.17.17188 PMC3218599

