# Supplementary material for: Attenuated RORC Expression in the Presence of EMT Progression in Somatotroph Adenomas following Treatment with Somatostatin Analogs Is Associated with Poor Clinical Recovery
Source: PLoS One. 2013 Jun 25;8(6):e66927. doi: 10.1371/journal.pone.0066927 (PMC3692554; doi:10.1371/journal.pone.0066927)
Supplement: Table S1 — Primer sequences used in PCR reactions. (DOCX) [file pone.0066927.s002.docx]

| **Genes** | **GenBank** | **Forward (5´to 3´)** | **Reverse (5´to 3´)** | **Size (bp)** |
| --- | --- | --- | --- | --- |
| CDH1 | NM_004360.3 | GCATTGCCACATACACTCTCTTCT | TCGGTTACCGTGATCAAAATCTC | 90 |
| RORC | NM_001001523.1  NM_005060_3 | CTCCCTGACAGAGATAGAGCACCT | CCCGGGAGAAGATGTTGGA | 113 |
| ITGAV | NM_001145000.1  NM_002210.3 | TGTGCCGCGCCTTCA | ACGAGAAGAAACATCCGGGAAG | 124 |
| CTTNBP2 | NM_033427.2 | CAAAAGAAGCTGGAAATGGAGAAG | TTTGACCAGCATCAGGACCAC | 123 |
| TIMP1 | NM_003254.2 | GCCCAGAGAGACACCAGAGAAC | AGCAACAACAGGATGCCAGAA | 71 |
| DOK6 | NM_152721.5 | AGCTTGGGATTTTCAGACGATG | ATTGTGCAGTTCAGTTACCTTATGAAAG | 134 |
| MBNL3 | NM_001170704.1  NM_001170703.1  NM_001170702.1  NM_001170701.1  NM_133486.2  NM_018388.3 | TCATCACTTGGTTCTTTTCCTATGAC | GTATTTGGTACAAGTTCTGCAGGAAC | 119 |
| NOX4 | NM_001143837.1  NM_001143836.1  NM_016931.3 | CAGATGTTGGGGCTAGGATTGT | CCTCGGAGGTAAGCCAAGAGT | 113 |
| VLDLR | NM_003383.3  NM_001018056.1 | GAAGAAAACTGTGGCAATATAACATGTAG | CCATCGCTGCAGTCATCCT | 110 |
| KCNIP3 | NM_013434.4 | TGCCTCGGCTGTGAAGTG | ACTTCCTTAGCCGGCTGCAT | 72 |
| WFDC1 | NM_021197.2 | CAGTCTTAGACTGGCTGGTGCAG | TCCTCCGTGGTGCTGCAC | 112 |
| NDRG1 | NM_001258433.1  NM_001258432.1  NM_001135242.1  NM_006096.3 | GTCCTTCAACAGTTTGGGCTGA | GGACAAGGCCCTCCACCAT | 112 |
| MMP16 | NM_0059414.4 | CAGAAGATATATGGTCCACCTGACAA | AGGCCGAGGAGGTTTTGG | 126 |
| MSN | NM_002444.2 | AGGTGGTGAAAACTATTGGCTTGAG | ACCTTCTTATTGAGTTTCAGCCAGG | 100 |
| PDK1 | NM_002610.3 | CATGGTGTTTGAACTTTTCAAGAATG | CCAGCGTGACATGAACTTGAATAG | 98 |
| POMC | NM_000939.2  NM_001035256.1 | GCTGGCCTTGCTGCTTCA | ATGCACTCCAGCAGGTTGCT | 102 |
| VAT1L | NM_020927.1 | CGTGGGTCAAGCTGTGGCT | TCTTTGATTGCTTCATGCTTGAAA | 93 |
| SLC24A2 | NM_001193288.2  NM_020344.3 | CCAAGCAAAGCCATCTGCA | CTCATGAGACTGTTGTGGAGGG | 108 |
| HCN1 | NM_021072.3 | CCTTACAGTGATTTCAGGTTTTACTGG | AATAATCCATGGTGTTGTTGTTTGC | 123 |
| ITPR2 | NM_002223.2 | TTGGGGTTAGTGGATGACAGATG | ATATCTGTTCATAGGGCACACCTTG | 111 |
| TMEM47 | NM_031442.3 | TGACCACCAGTACTACCTGTCGTT | GTAGCAATCTGCCAATCGCTG | 105 |
| GALNT5 | NM_014568.1 | AGACCTGCTGGATGTGCAGAG | TTGATGACACTGTGAACAGATCTCAG | 119 |
| SEMA5A | NM_003966.2 | GCTCCTCGCACCTGTGTTG | AGTGCCAAGGAGAGAGAGGATG | 116 |
| RPH3A | NM_001143854.1  NM_014954.3 | AATCGGACGCCTGGTGGA | CACAGGCAGAGCCCAGCA | 113 |
| NDNF | NM_024574.3 | TTAAGGATGGTGCTGCTCCAC | ATGCCTTGTCCCGGATCTG | 121 |
| SSTR2 | NM_001050.2 | AGCAAAGATGTCACACTGGATCC | CCATGGCTGCTTTTCAGTCTTAG | 103 |
| β-ACTIN | NM_001101.3 | AGGCACCAGGGCGTGAT | TCGTCCCAGTTGGTGACGAT | 128 |
| GAPDH | NM_001256799.1  NM_002046.4  NM_017008.3 | CCAAGGTCATCCATGACAACTT | AGGGGCCATCCACAGTCTT | 93 |
